# IMPACT OF HOME HEALTH VALUE-BASED PURCHASE MODEL ON OWNERSHIP OF HOME HEALTH AGENCY: A LONGITUDINAL ANALYSIS

**DOI:** 10.1093/geroni/igad104.2920

**Published:** 2023-12-21

**Authors:** Chenjuan Ma

**Affiliations:** New York University, New York City, New York, United States

## Abstract

Home health care serves over 5 million Americans “aging in place”. In January 2016, home health value-based purchasing model (HHVBP) was introduced and piloted in 9 states in the US with a purpose of improving care quality and efficiency, and expanded nationwide in 2023. This study aimed to examine the impact of HHVBP on business operation of home health agencies (HHA), including ownership changes. This is a longitudinal study using 7 years (2013-2019) of data from two national data sources. A dummy variable was used to indicate whether an HHA is in a state with HHVBP or not. Ownership measures included both the type and changes of ownership between 2013-2017. A total of 14,334 HHAs (95,137 agency-years) were included in analysis. Of the 14,334 agencies, about 19% were in HHVBP states, 88% continued business in all study years, 1679 were new agencies and 49 agencies discontinued business. In HHVBP states, 363 out of 2781 (13%) were newly added, compared to 1316 out of 11,553 (11%) in non-HHVBP states. Agencies in HHVBP states were more likely to be public agencies (e.g., 6.08% vs. 4.97% in 2013; 5.62% vs. 4.60% in 2018), though it slightly decreased over time; and experience changes in ownership and particularly the years after the implementation of HHVBP (e.g.,2.44% vs. 2.38% in 2013; 7.03% vs. 4.39% in 2018). The HHVBP can have a significant impact on the operation and ownership type of home health agency, which in turn may impact availability and quality of home health care.

